# Interpretable meta-learning of multi-omics data for survival analysis and pathway enrichment

**DOI:** 10.1093/bioinformatics/btad113

**Published:** 2023-03-02

**Authors:** Hyun Jae Cho, Mia Shu, Stefan Bekiranov, Chongzhi Zang, Aidong Zhang

**Affiliations:** Department of Computer Science, University of Virginia, United States; Department of Computer Science, University of Virginia, United States; Department of Biochemistry and Molecular Genetics, University of Virginia, United States; Center for Public Health Genomics, University of Virginia, United States; Department of Public Health Sciences, University of Virginia, United States; Department of Computer Science, University of Virginia, United States

## Abstract

**Motivation:**

Despite the success of recent machine learning algorithms’ applications to survival analysis, their black-box nature hinders interpretability, which is arguably the most important aspect. Similarly, multi-omics data integration for survival analysis is often constrained by the underlying relationships and correlations that are rarely well understood. The goal of this work is to alleviate the interpretability problem in machine learning approaches for survival analysis and also demonstrate how multi-omics data integration improves survival analysis and pathway enrichment. We use meta-learning, a machine-learning algorithm that is trained on a variety of related datasets and allows quick adaptations to new tasks, to perform survival analysis and pathway enrichment on pan-cancer datasets. In recent machine learning research, meta-learning has been effectively used for knowledge transfer among multiple related datasets.

**Results:**

We use meta-learning with Cox hazard loss to show that the integration of TCGA pan-cancer data increases the performance of survival analysis. We also apply advanced model interpretability method called DeepLIFT (Deep Learning Important FeaTures) to show different sets of enriched pathways for multi-omics and transcriptomics data. Our results show that multi-omics cancer survival analysis enhances performance compared with using transcriptomics or clinical data alone. Additionally, we show a correlation between variable importance assignment from DeepLIFT and gene coenrichment, suggesting that genes with higher and similar contribution scores are more likely to be enriched together in the same enrichment sets.

**Availability and implementation:**

https://github.com/berkuva/TCGA-omics-integration.

## 1 Introduction

Traditionally, survival analysis has usually been done by means of statistical analyses that aim to identify the distributions of event times and the statistical properties of the estimated parameters. In comparison, machine learning algorithms aim to produce more accurate survival prediction by combining established statistical methods with machine learning methods. Yet, a high ratio of missing survival information, data sparsity, missing features, and labels can cause significant loss of performance even with some of the most recent machine learning algorithms. In particular, deep neural network machine learning approaches, which have shown promising results in various aspects of biomedical studies, require a large amount of well-organized and complete data for training, which is rare in biomedical settings.

Recently, high throughput techniques together with the continuously decreasing cost of sequencing have resulted in a wide availability of multiomics data. Because different molecular processes starting on DNA and resulting in protein products are intricately interconnected, omics data derived from different stages of gene and protein expression are likely to contain complementary information. However, the explicit relationships among multiomics data are complex and remain poorly understood. Moreover, many omics data suffer from the problem of ‘big P, small N’—having significantly higher number of features (P) than the number of data samples (N). This makes integrating and interpreting multiomics data even more challenging.

As a result of those challenges, many existing works on survival analysis use a single omics dataset, such as clinical or transcriptomics (Spooner *et al*. 2020; Srujana *et al*. 2022). Only recently, advanced machine learning-based approaches including transfer learning and representation learning have been applied to alleviate the ‘big P, small N’ problem in omics datasets (Kim *et al*. 2020; Qiu *et al*. 2020). Although such approaches have shown promising advances in survival analysis, the integration of multiple omics data is likely to further improve the performance of advanced machine learning applications on survival analysis and related tasks due to the unique feature sets in each omics dataset. However, the lack of standards and applications for omics data integration challenge the attempt for multi-omics integration for machine learning applications.

Meta-learning methods have been adopted widely to handle multiple related tasks (Finn *et al*. 2017). These learning-to-learn methods are first trained on a distribution of related tasks before being fine-tuned and evaluated on target tasks. It has been shown that meta-learning methods often require fewer samples than more traditional deep learning models (Triantafillou *et al*. 2020; Gevaert 2021). As a result, we propose a meta-learning approach that uses multi-omics datasets to train a hazard predictive model for cancer survival analysis. In particular, we use transcriptomics (RNA-seq), proteomics, and clinical datasets from The Cancer Genome Atlas (TCGA). Compared with most prior studies that rely exclusively on one omics dataset for pan-cancer analysis, we apply our meta-learning model on various combinations of integrated omics datasets.

Additionally, we analyze the benefit of multi-omics data integration on identifying differentially expressed genes for deep learning-based survival analysis. Given the black-box nature of deep neural network-based algorithms, the convenience of applying them leads to difficulties in interpretability. To address this, we apply an advanced variable importance analysis method for deep learning, DeepLIFT (Deep Learning Important FeaTures) (Shrikumar *et al*. 2017), and compare pathway enrichment for transcriptomics and multi-omics data. More specifically, each input gene to our meta-learning model is assigned a DeepLIFT contribution score. We then set a cutoff score and assign genes with above-the-cutoff-score as the input to g: Profiler (Raudvere *et al*. 2019) to identify enriched pathways. Additionally, we use Gene Set Enrichment Analysis (GSEA) (Subramanian *et al*. 2020) for pathway enrichment analysis from a ranked list of genes’ DeepLIFT scores. Finally, we use a neural network extension of the Cox regression model and the protein enrichment database STRING (Jensen *et al*. 2009) to show that genes with similar DeepLIFT contribution scores are statistically more likely to be enriched together.

## 2 Related work

### 2.1 Multi-omics data integration

Statistical approaches have been applied for using integrated pan-cancer multi-omics data. For instance, (Mankoo *et al*. 2011) integrated gene expression, miRNA expression, copy number alteration, and DNA methylation data to predict time to recurrence, survival, and risk predictions in ovarian cancer using multivariate Cox Lasso. iCluster (Shen *et al*. 2009) is a tumor subtype estimation method that works by joint latent variable modeling for integrative clustering. It fits a regularized latent variable model based clustering to generate an integrated cluster assignment based on joint inference across multiple genomic data types, such as copy number, gene expression, and DNA methylation. PARADIGM (Vaske *et al*. 2010; Subramanian *et al*. 2020) infers the activities of patient-specific biological pathways from multi-omics data using Bayesian factor graphs. MANCIE (Zang *et al*. 2016) uses a Bayesian-supported principal component analysis-based approach to address the noises and biases that arise in high-dimensional genomic data integration. Various forms of non-negative matrix factorization (NMF) methods have also been introduced for identifying modules of correlated multi-omics data, sample clustering and subtype discovery (Lee and Seung 1999; Zhang *et al*. 2012; Yang and Michailidis 2016; Chalise and Fridley 2017).

Various machine learning approaches have been adopted for multi-omics data. MOLI (Sharifi-Noghabi *et al*. 2019) and DeepMO (Lin *et al*. 2020) implement subnetworks on each omics dataset and concatenate the obtained deep features for prediction of drug activity (Picard *et al*. 2021). Ma and Zhang (2018) integrated gene expression, miRNA expression, and DNA methylation data and implemented Multi-view Factorization AutoEncoder (MAE) to predict target clinical variables.

The recent development of data repositories such as BioGRID (Oughtred *et al*. 2021) and STRING (Szklarczyk *et al*. 2021) have allowed the integration of *a priori* information and inter-omics relationships for model development and interpretation. While new datasets are constantly being added to them, the integration of features that are relevant but vastly different such as demographics, genes, and mutation significance remains challenging, and there exists no standards for feature engineering and model selection for such multi-omics data, especially for pan-cancer data.

### 2.2 Pan-cancer data analysis and molecular pathways

Numerous methods of pan-cancer analysis have been conducted for different cancer types, such as identifying molecular subtypes significant for clinical studies (Sohn *et al*. 2017; Oh *et al*. 2020), classifying genes with high morbidity correlation (Teo *et al*. 2019), and building next-generation sequencing (NGS)-based diagnostics for lung cancer (Chun *et al*. 2019).

Traditional machine learning algorithms, such as random forests and support vector machines, have shown promising performance in various areas of pan-cancer data analysis, including cancer type classification and detection of biomarkers (Furey *et al*. 2000; Toth *et al*. 2019; Liñares-Blanco *et al*. 2020).

More recently, non-parametric machine learning models, particularly deep neural networks, have been applied to more complex pan-cancer data analysis, such as multi-omics cancer type classification and survival prediction. For example, Kong *et al*. (2021) used an autoencoder and a backward propagation-based neuron importance assignment method to capture nonlinear relationships among genes to develop a robust cancer type classification that outperformed L1000 expression profiling, a reduced representation of ∼1000 genes whose expression is highly representative (Subramanian *et al*. 2017). Withnell *et al*. (2021) trained a variational autoencoder to capture the dense genetic relationships for each cancer type to classify different cancer types.

Kim *et al.* (2020) applied transfer learning through a variational autoencoder, transferring trained encoder parameters to a Cox-hazard based prediction network. Their VAECox model improved regularization-based Cox Hazard models on 7 out of 10 cancer types tested. They applied Pearson’s correlation coefficient on the neurons in the hidden layers of their encoder network with the most variance for determining the most important genes for breast cancer. This led them to identify cancer-related genes with existing literature support. A recent work on cancer type classification found that meta-learning outperformed more traditional machine learning-based approaches (Chou *et al*. 2020). Qiu *et al.* (2020) used a meta-learning-based Cox module on various cancer types to train a quickly adaptive model, learning the common latent information across the training cancer types. They showed that their model achieved notable testing performance increases compared to direct learning and transfer learning methods. They used gradient-based feature importance followed by GSEA to determine the important molecular pathways for each of their target cancer types.

## 3 Materials and methods

### 3.1 Data and preprocessing

The Cancer Genome Atlas Program, or TCGA, is a landmark cancer program that provides public cancer genomics datasets covering 33 cancer types. We use transcriptomics (RNA-seq), proteomics, and clinical datasets. These datasets contain information in different molecular stages: proteins are translated from RNA, and the functional mechanisms of cells are determined from proteins, leading to various clinically relevant outcomes.

For the clinical and the proteomics datasets, we imputed missing numeric feature values using the k-nearest neighbors (KNN) method, with k = 1. We used label encoding to encode categorical features. In the clinical dataset, we manually removed the feature that contains patients’ vital status, which has a high correlation with their survival status. The transcriptomics dataset required no imputation. We then applied z-score transformation for each dataset after imputation. Finally, in order to remove features with high correlation to each other, we filtered out features with over 0.7 Pearson correlation to at least one other feature.

Post-imputation, scaling, and feature engineering, the transcriptomics, the clinical, and the proteomics datasets in the TCGA comprised 11 068, 8490, and 5853 patients, respectively, and 13 960, 10 039, and 227 features. To integrate the multiple omics datasets, we reapplied z-scale transformation and filtered out features with a correlation of over 0.7 to another feature in the integrated dataset.

Although there are 33 cancer types in TCGA, the variations among them can result in high bias and noise when all are used to construct the input for the learning model. Therefore, we carefully selected 17 cancer types that satisfy the following two conditions: (1) sufficient number of patients in all three datasets and (2) long follow-up times. We reviewed (Liu *et al*. 2018), a comprehensive study of the quality of TCGA clinical data, and selected ACC, BRCA, CESC, COAD, ESCA, GBM, KIRP, LGG, LUSC, OV, PAAD, STAD, UCEC, and UCS as source tasks and BLCA, HNSC, and LUAD as target tasks. The separation of train and evaluation data was chosen arbitrarily. Even though LUAD and LUSC are both lung-related cancer types, we do not merge them into one lung cancer type like (Qiu *et al*. 2020) did because they are known to be transcriptomically different (Relli *et al*. 2019). There were 4647 patients whose data existed in all three omics datasets. We reserved 30 samples from each cancer type as the query set for model evaluation on each respective cancer type and used the rest as the support set for training the model. The size of support and query sets for each cancer type, as well as the proportion of censored patients, are shown in Table 1. A patient’s data is censored if the patient left the TCGA study before death; hence, their time-to-death is unknown.

**Table 1.** Number of support (training) and query (evaluation) sets for source and target cancer types.

Cancer type	No. of support set	No. of query set	% censored
ACC	16	30	69.6
BRCA	840	30	86.1
CESC	139	30	81.7
COAD	290	30	77.8
ESCA	95	30	64.8
GBM	27	30	29.8
KIRP	175	30	85.4
LGG	395	30	77.2
LUSC	286	30	59.2
OV	189	30	35.2
PAAD	68	30	42.9
UCS	18	30	35.4
UCEC	361	30	82.6
STAD	302	30	60.2
BLCA	308	30	54.7
HNSC	308	30	50.0
LUAD	320	30	60.0

Thirty samples from each cancer type are reserved for the query set, which is used to evaluate the model. The percentage of censored patients varies for each cancer type.

### 3.2 Optimization

Driven by statistical analysis and the existence of censored data, non-parametric methods have become more popular over parametric methods due to the following reasons: (i) fewer assumptions and (ii) no distributional constraints on survival times.

Given many collected features in a dataset, Cox regression models determine which features conduce to a probability of survival. By taking the natural logarithm of the hazard function, we obtain $\text{log}\;h(t,x)=\text{log}\;h_{0}(t)+b^{T}x$, where $h_{0}$ is the baseline hazard, and *x* is a set of covariates. This means the hazard ratio does not depend on the baseline hazard and remains constant. This leads to one of the most widely used time-to-event analysis, Cox Proportional Hazard (Cox-PH) method. Cox-PH is a semi-parametric model that calculates the probability of an event occurring in relation to a time variable.

The Cox hazard loss $L_{C}$ is used to find the optimal partial log-likelihood estimates of the model parameters β: where for sample *i*, $C_{i}=1$ indicates the occurrence of the event of interest, $X_{i}$ is the representation of the dependent variables, and $Y_{i}$ is the time to event occurrence.


(1) $$L_{C}(\beta )=\sum_{i:Ci=1}\left(X_{i}\beta -\log\sum_{j:Y_{j}\geq Y_{i}}e^{X_{j}\beta }\right)$$

Recently, the Cox-PH model has been combined with various forms of artificial deep neural networks, allowing flexible model selection and thereby enhancing the performance compared to regularized Cox-PH methods, such as in Kim *et al*. (2020) and Qiu *et al*. (2020). To study whether the underlying information contains complementary survival data in spite of weak correlations among the omics datasets, we implemented a meta-learning model and applied it on the transcriptomics and on various integrated omics datasets with Cox hazard loss.

### 3.3 Meta-learning for survival analysis

**Figure 1.**
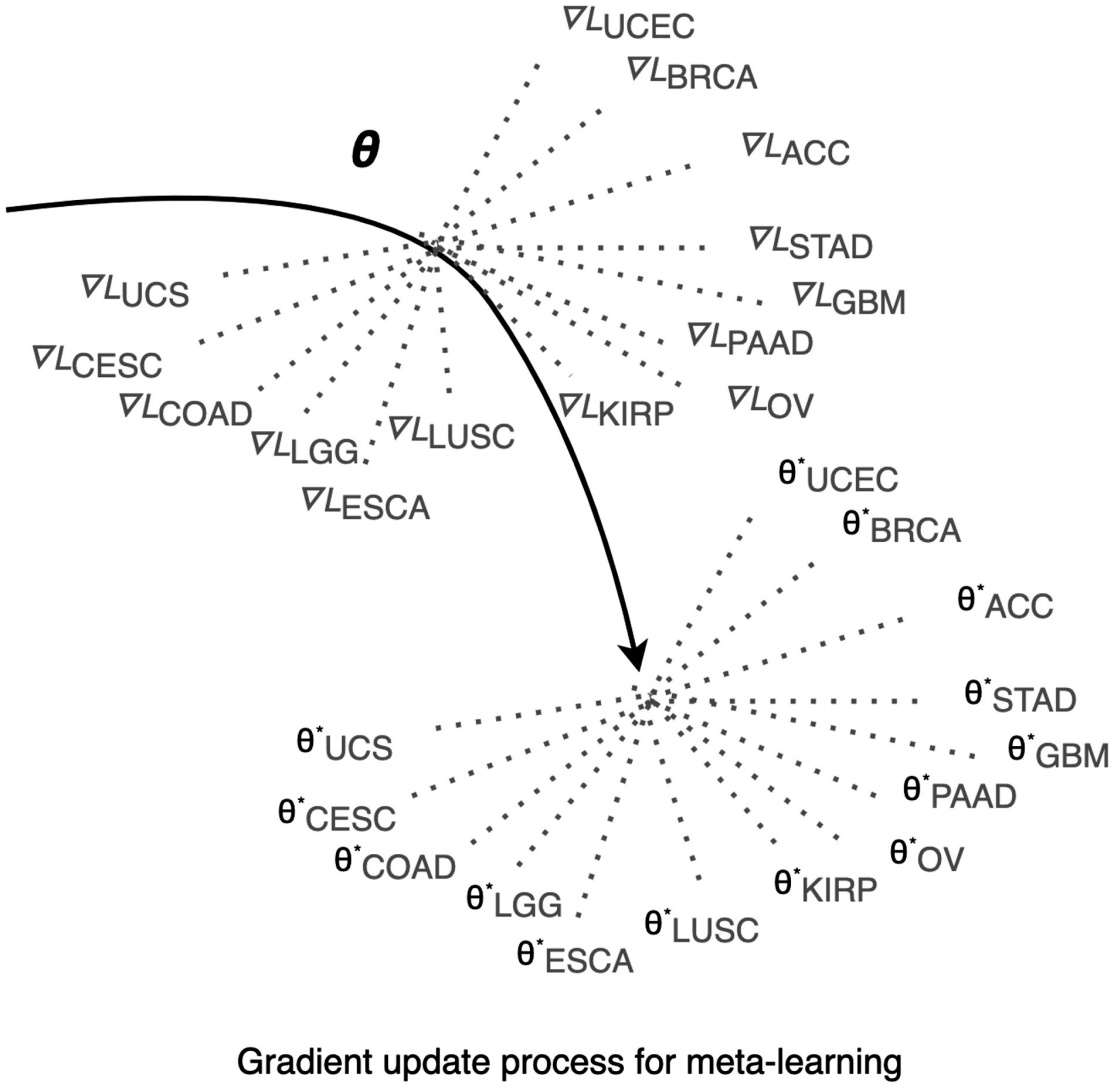
Our meta-learning model’s parameters θ are trained over a distribution of source cancer types, and the corresponding loss *L* are generated. θ are optimized by gradient descent, or back-propagation of $\nabla L_{{\text{task}}_{i}}$

Meta-learning is an algorithm that allows quick adaptation across multiple similar datasets. It is first trained on related source data, followed by fine-tuning and evaluation on target tasks. Back-propagation of the gradients generated from the loss incurred from each task leads to the optimal values of θ, as illustrated in Fig. 1. Its parameters θ are generically trained to minimize respective cox hazard loss, *L*, allowing quick adaptation to new tasks.

The meta-learning model used in our survival analysis is Model-Agnostic Meta-Learning for Fast Adaptation of Deep Networks (MAML) (Finn *et al*. 2017). MAML is a task-agnostic algorithm for meta-learning that trains model parameters such that a small number of gradient updates will lead to fast learning on a new task. MAML’s learning (training) process is largely divided into two parts: inner and outer loop. The inner loop trains on source tasks, and multiple inner loop training steps together update the parameters in the outer loop so that they are generalized to adapt to new (target) tasks quickly. Within each task, the data are split into support and query sets. The model is trained on the support sets and its performance is evaluated on each task using the query set. We split the support and query sets so that the query set contains 30 samples, and the rest is used as the support set.

Our MAML model, we used a four-layered feed-forward neural network. For nonlinear activation, we used ReLU. For inner loop training, we use SGD optimizer and a learning rate of 0.01. For outer loop training, we use Adam optimizer and a learning rate of 0.0001. Finally, we compare our model’s performance with the performance of direct learning with 5-fold cross validation.

### 3.4 Identifying important molecular pathways with explanations

As detailed below, the variable importance assignment approaches that we applied produce scores associated with our input variables. This includes scores associated with thousands of genes. Consequently, to assess the significance and cancer relevance of the gene sets that are driving survival prediction, we took two common pathway enrichment approaches: (1) applying a variable importance score cutoff and inputting the resulting gene list into a pathway enrichment tool and (2) using all annotated genes in the genome and their associated scores to arrive at enriched pathways.

For the first approach, we used g: Profiler to find functionally enriched pathways on the limited gene list. For the second approach, we performed gene set enrichment analysis (GSEA) by using a ranked list of genes with scores. The selection of genes and the assignment of scores were done by applying DeepLIFT (Deep Learning Important FeaTures) to our meta-learning model that estimates the patients’ probability of survival by optimizing cox-loss. DeepLIFT is a recently developed method for gaining interpretability for deep neural networks. Interpretability methods applicable for deep neural networks such as DeepLIFT largely consist of two methods: forward propagation and backward propagation methods. Forward propagation methods, such as (Alipanahi *et al*. 2015; Zhou and Troyanskaya 2015), generate permutations of input features to measure the impact of each feature on the output. This is highly inefficient due to exponential growth of possible permutations with a large feature set. More recently, backward propagation interpretability methods such as DeepLIFT have been adopted widely for model intelligibility.

DeepLIFT first assigns reference values, or null values, to the input neurons in the network. It forward propagates the difference between the value and its reference value. Then, it calculates how much each input affects its subsequent neuron, or it ‘blames’ the difference from reference of the output on the difference from reference of the input. This difference is then allocated (or blamed) to each input neuron by back-propagation, assigning contribution scores to all neurons in the network. Related interpretability methods, such as integrated gradients (Sundararajan *et al*. 2017) and SHAP (SHapley Additive exPlanations) (Lundberg and Lee 2017), calculate variable contribution in similar back propagation-based approaches.

### 3.5 Gene-set co-enrichment

We also studied whether DeepLIFT importance analyses are correlated with gene coenrichment using STRING (Jensen *et al*. 2009), a database for protein–protein interaction networks and functional enrichment. Specifically, we used the meta-learning model from survival analysis that was trained on the source cancer types and fine-tuned to each target cancer type on the transcriptomics data.

**Figure 2.**
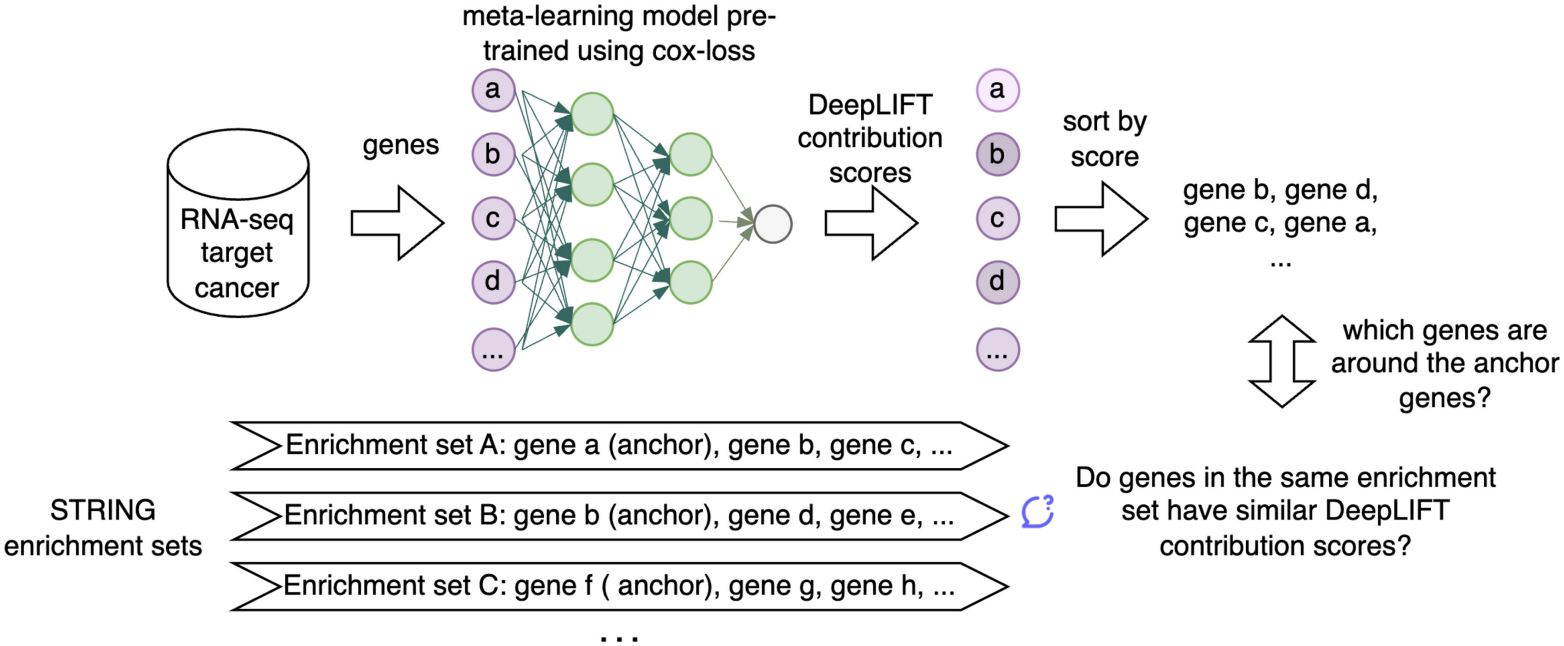
The process of studying the correlation between genes with similarly high DeepLIFT contribution scores and their likelihood of being enriched together in the same STRING enrichment sets

We analyzed the degree of correlation between genes with similar contribution scores and genes’ coenrichment in functional sets in STRING. To test this, we sorted the genes by their contribution scores by applying DeepLIFT to the fine-tuned meta-learning model and checked how the genes in the same enrichment set are clustered together in the sorted list compared with how they are in a randomly sorted list. In each enrichment set, we chose the first gene as the anchor gene and checked whether the surrounding genes in a certain window size around it in the sorted and unsorted list are in the same enrichment set. We generated STRING enrichment sets using genes in the 50th, 70th, and 90th percentiles of DeepLIFT scores. This process is illustrated in Fig. 2.

Being able to learn gene coenrichment information via importance analyses without prior knowledge indicates the functional, pathological, and semantic relationships among genes that are captured by the model. This experiment is conducted using the transcriptomics genes for the three target cancer types: BLCA, HNSC, and LUAD.

## 4 Results

### 4.1 Evaluation metrics

In this section, we explain how we evaluate the performance of survival analysis by meta-learning, enriched pathways, and gene coenrichment.

Cox loss-based models predict the probability of survival given a time step. Concordance index (C-index) measures the relative order of predicted patients’ hazard times to the actual order. Hence, it is one of the most widely used evaluation metrics for Cox loss-based survival analyses. It is calculated by dividing the number of concordant pairs by the sum of concordant and discordant patient pairs. A pair of patients is concordant if the predicted order of the event is the true order; otherwise, it is a discordant pair. We evaluated the C-index of the meta-learning model on the target tasks on two conditions: using transcriptomics dataset alone and using different combinations of the three datasets.

A metric for evaluating the error of survival prediction includes the integrated Brier score. It is calculated by the mean squared differences between predicted and ground truth survival probabilities at a given time step. Therefore, we use integrated Brier score (Clarke and Wallis 2008) to evaluate the model’s survival probabilities for different combinations of the three datasets.

Given a list of enriched genes and attribution scores, GSEA calculates the number of statistically significantly enriched molecular pathways differentially expressed between survived and deceased patients. The higher the attribution scores are for the genes that are coenriched in a pathway for a group of patients, the more statistically significant the pathways. However, there exists no standard for objectively comparing the goodness of enriched pathways. Therefore, we provide literature support for a few selected pathways, supporting their enrichment status for cancer.

As a follow-up study for interpreting DeepLIFT scores and pathway analysis, we compare how genes with similar DeepLIFT attribution scores appear in the same STRING enrichment sets with how genes with different attribution scores do. We queried STRING with genes with the highest attribution scores in the 50th, 70th, and 90th percentiles to observe correlation between attribution scores and gene coenrichment. Finally, we studied how the distribution of categories of enriched pathways change with different percentiles of DeepLIFT attribution scores.

### 4.2 Meta-learning for survival analysis

In Table 2, we show the performance of meta-learning on individual, paired, and integrated omics datasets on the target cancer types.

**Table 2.** 95% confidence interval for concordance index achieved by metalearning on using individual and integrated datasets.

Omics	No. of features	BLCA	HNSC	LUAD
Direct learning ($D_{c,t,p}$)	24 218	0.66±0.01	0.62±0.01	0.50±0.01
Transcriptomics ($M_{t}$)	13 960	0.74±0.03	0.70±0.03	0.62±0.01
Clinical ($M_{c}$)	10 039	0.75±0.02	0.77±0.02	0.71±0.02
Proteomics ($M_{p}$)	227	0.58±0.03	0.60±0.04	0.53±0.03
Clinical and transcriptomics ($M_{c,t}$)	23 994	0.75±0.01	0.77±0.01	0.72±0.05
Transcriptomics and proteomics ($M_{t,p}$)	13 724	0.64±0.03	0.63±0.02	0.66±0.04
Proteomics and clinical ($M_{p,c}$)	10 266	0.66±0.02	0.79±0.01	**0.73 ± 0.01**
Integrated omics ($M_{c,t,p}$)	24 218	**0.79 ± 0.04**	**0.84 ± 0.01**	**0.73 ± 0.04**

Dimensions indicate the number of features in each omics dataset(s) after removing features with 0.7 Pearson correlation. Higher values represent better performance.

For convenience, we denote our meta-learning model *M* trained on each dataset *d* as $M_{d}$, such as $M_{c}$, $M_{t}$, $M_{p}$, $M_{c,t}$, $M_{t,p}$, $M_{p,c}$, and $M_{c,t,p}$, where *c*, *t*, and *p* indicate the clinical, the transcriptomics, and the proteomics, respectively, and any combination of two or all three of these subscripts indicates dataset integration. Similarly, we denote the direct learning baseline model trained on *c*, *t*, *p* as $D_{c,t,p}$. The direct learning method was trained on all training data simultaneously. Its performance was then evaluated on the 30 query set of each target cancer type. Its c-index scores were 0.66, 0.62, and 0.50 on BLCA, HNSC, and LUAD, respectively.

Qiu *et al*. (2020) described how meta-learning performance is superior to direct learning as well as regular transfer learning when all models were trained on the transcriptomics dataset. The results from our experiment extend their analysis; on BLCA, HNSC, and LUAD, $M_{t}$ recorded 0.74, 0.70, and 0.62 c-index, outperforming $D_{c,t,p}$.

Compared with $M_{c}$ or $M_{t}$, $M_{p}$ performed worse, with 0.58 on BLCA, 0.60 on HNSC, and 0.53 on LUAD. We believe this to be due to the relatively small number of features compared with the other two datasets.

We then ran survival analysis on pairwise integrated datasets. To perform data integration, we merged the features from each pair of datasets and removed features with higher than 0.7 Pearson correlation to at least one other feature. The resulting dimensions after integrating and removing the feature were 23 994 for the clinical and the transcriptomics datasets, 13 724 for the proteomics and the transcriptomics datasets, and 10 266 for the proteomics and the clinical datasets.

$M_{c,t}$ performed similarly to $M_{c}$ but outperformed $M_{t}$ across all target cancer types, scoring 0.75 on BLCA, 0.77 on HNSC and 0.72 on LUAD. We analyzed which significantly mutated genes were associated with the highest DeepLIFT scores and validated their direct or indirect contributions to each cancer type by existing research support: COPZ2, MED23, SUZ12, CYP4B1, NFE2L2 for BLCA (Imaoka *et al*. 2000; Fan *et al*. 2014; Sharma *et al*. 2019; Yi *et al*. 2021; Zhang *et al*. 2022); JAK2 for HNSC (William *et al*. 2021); 22q12.1, SNRPD3, SLC14A2, RCVTB2, ITGAM, Mrc1, SETD2 for LUAD (Neville *et al*. 1995; Wang *et al*. 2014; Tariq *et al*. 2017; van Olst *et al*. 2017; Wang *et al*. 2017; Bai *et al*. 2019; Chang *et al*. 2021; Gao *et al*. 2021; Li *et al*. 2021; Larroquette *et al*. 2022).

$M_{t,p}$ experienced a decreased performance on BLCA and HNSC compared with $M_{t}$ but an increase on LUAD by scoring 0.64, 0.63, and 0.66, respectively. Compared with $M_{c}$, $M_{c,p}$ experienced a decreased performance on BLCA but meaningful increases on HNSC and LUAD with c-index of 0.66, 0.79, and 0.73, respectively.

Finally, we expanded our survival analysis by integrating all three datasets. As we did to the paired datasets, we also removed features with 0.7 or higher Pearson correlation, resulting in 24 218 features. $M_{c,t,p}$ achieved the best c-index on all cancer types, with 0.79, 0.84, and 0.73 scores respectively. The fact that $M_{c,t,p}$ has performed better than $M_{c,t}$ tells us that the addition of the proteomics dataset was a contributor to the increased performance. This shows that although the integration of omics datasets can lead to improved performance on survival analysis, it can be highly dependent on the types of omics datasets involved as well as the specific cancer types that the model is applied to.

Our results summarized in Table 2 show that meta-learning can perform superior to direct learning and that the integration of certain omics dataset can lead to increased performance, which can depend on the types of integrated omics dataset and on the cancer type. Moreover, it is noteworthy that the performance of survival analysis can significantly rely on several factors, including the data preprocessing methods, the selection of source and target cancer types, and the quantity of samples used for fine-tuning the model for each target cancer. These results are mostly aligned with the integrated brier scores summarized in Table 3.

**Table 3:** 95% confidence interval for integrated Brier scores.

Omics	BLCA	HNSC	LUAD
Transcriptomics ($M_{t}$)	0.19±0.02	0.23±0.01	0.27±0.01
Clinical ($M_{c}$)	0.11±0.01	0.19±0.01	0.26±0.01
Proteomics ($M_{p}$)	0.30±0.02	0.33±0.01	0.37±0.01
Clinical and transcriptomics ($M_{c,t}$)	0.26±0.02	0.26±0.01	0.25±0.01
Transcriptomics and proteomics ($M_{t,p}$)	0.29±0.02	0.30±0.01	0.25±0.01
Proteomics and clinical ($M_{p,c}$)	0.28±0.01	0.20±0.01	0.20±0.03
Integrated omics ($M_{c,t,p}$)	0.14±0.02	0.12±0.01	0.16±0.01

Lower values represent better performance.

### 4.3 Identifying important molecular pathways with explanations

We tried four DeepLIFT reference values: the median and the mean feature values for deceased patients and survived patients individually. The median feature values for deceased patients yielded higher number of enriched molecular pathways.

In Table 4, we show statistically enriched KEGG (Kanehisa and Goto 2000) and WikiPathways (Pico *et al*. 2008) using g: Profiler and GSEA. For selecting the query genes against the background genes for g: Profiler, we filtered out the genes with less than the mean scores from each target cancer type. On the other hand, we assigned every gene and its corresponding DeepLIFT contribution scores as input to GSEA. In order to correctly show statistical significance for multiple hypothesis testing, we show pathways with adjusted p values less than 0.05 for g: Profiler and FDR q-values less than 0.25 for GSEA.

**Table 4.** Significantly enriched WikiPathways (WP) and KEGG pathways determined by DeepLIFT found using GSEA and g: Profiler.

Pathway	Data	Cancer	Significance
(KEGG-g: Profiler) pathways in cancer	RNA-seq	BLCA	1.216e${}^{-14}$
(WP-g: Profiler) VEGFA-VEGFR2 signaling pathway	RNA-seq	BLCA	1.216e${}^{-14}$
(KEGG-GSEA) intestinal immune network for IGA production	RNA-seq	BLCA	0.127
(KEGG-g: Profiler) pathways in cancer	RNA-seq	BLCA	1.189e^−7^
(WP-GSEA) BDNF TRKB signalling	Multi-omics	BLCA	0.170
(WP-g: Profiler) oxidation by cytochrome P450	Multi-omics	BLCA	2.599e^−9^
(KEGG-GSEA) type II diabetes mellitus	Multi-omics	BLCA	0.120
(KEGG-g: Profiler) ABC transporters	Multi-omics	BLCA	2.249e^−17^
(WP-GSEA) methionine *de novo* and salvage pathway	RNA-seq	HNSC	0.044
(WP-g: Profiler) head and neck squamous cell carcinoma signaling pathway	RNA-seq	HNSC	3.950e^−2^
(KEGG-GSEA) cytosolic DNA sensing pathway	RNA-seq	HNSC	0.044
(KEGG-g: Profiler) NF-kappa B signaling pathway	RNA-seq	HNSC	1.860e^−6^
(WP-GSEA) interactions between immune cells and micrornas in tumor microenvironment	Multi-omics	HNSC	0.226
(WP-g: Profiler) DNA repair pathways, full network	Multi-omics	HNSC	2.6e^−5^
(KEGG-GSEA) arginine and proline metabolism	Multi-omics	HNSC	0.064
(KEGG-g: Profiler) TNF signaling pathway	Multi-omics	HNSC	6.0e^−5^
(WP-GSEA) interactions between immune cells and micrornas in tumor microenvironment	RNA-seq	LUAD	0.171
(WP-g: Profiler) TLR signaling related to MyD88	RNA-seq	LUAD	3.736e${}^{-2}$
(KEGG-GSEA) nicotinate and nicotinamide metabolism	RNA-seq	LUAD	0.032
(KEGG-g: Profiler) metabolic pathways	RNA-seq	LUAD	5.090e^−10^
(WP-g: Profiler) VEGFA-VEGFR2 signaling pathway	Multi-omics	LUAD	5.235e^−10^
(KEGG-GSEA) biosynthesis of unsaturated fatty acids	Multi-omics	LUAD	0.191
(KEGG-g: Profiler) small cell lung cancer	Multi-omics	LUAD	1.697e^−2^

Multi-omics indicates integration of transcriptomics, clinical, and proteomics datasets. Significance values are FDR *q*-values <0.25 for GSEA and .05 adjusted *P*-values for g: Profiler.

For a number of pathways identified in Table 4, we identified existing literature support for their correlation with cancer. For example, DNA repair pathways are closely linked to cancer as they are responsible for maintaining genetic stability and integrity of DNA and cells (Alhmoud *et al*. 2020; Huang and Zhou 2021). When DNA repair pathways are disrupted, it can lead to mutations that can cause cancer. Additionally, some cancer cells are able to survive DNA damage caused by chemotherapy treatments due to their ability to repair the damage to their DNA (Kiwerska and Szyfter 2019). Inhibitors of certain DNA repair pathways can be used to further increase the effectiveness of chemotherapy treatments (Li *et al*. 2021).

The VEGF-VEGFR2 signaling pathway plays a critical role in the development and progression of cancer. Vascular endothelial growth factor (VEGF) stimulates angiogenesis (the formation of new blood vessels), which is necessary for tumors to grow and spread (Shibuya 2011). Vascular endothelial growth factor receptor 2 (VEGFR2) is the primary receptor for VEGF and is overexpressed in many types of cancer, leading to an increase in angiogenesis and tumor growth. Inhibitors of this pathway, such as tyrosine kinase inhibitors, have been developed and used as cancer treatments with varying degrees of success (García-Caballero *et al*. 2022).

Similarly, research suggests that oxidation by cytochrome P450 and ABC transporters are also related to cancer. Specifically, cytochrome P450 is a group of enzymes that are involved in the metabolism of drugs, hormones, and other substances in the body. They are also involved in the metabolism of carcinogens, which are substances that can cause cancer (Elfaki *et al*. 2018). Moreover, genetic polymorphisms of cytochrome P450 can lead to changes in its activity, which in turn can increase the risk of cancer. ABC transporters can act as a mechanism for multi-drug resistance (MDR). ABC transporters contribute to MDR in cancer by actively pumping chemotherapy drugs out of the cancer cells, reducing their effectiveness (Choi 2005).

TLR4 signaling pathway is involved in the development and progression of cancer and has been linked to inflammation and tumorigenesis (Chen *et al*. 2018). Activation of TLR4 can promote the growth and survival of cancer cells by signaling pathways that promote cell proliferation and suppress apoptosis (Huang *et al*. 2008). TLR4 can also induce angiogenesis, the formation of new blood vessels, which is important for tumor growth and spread. Additionally, TLR4 has been shown to be involved in the activation of immune cells and regulation of the immune response, which can impact the progression of cancer (Li *et al*. 2017). TLR4 has therefore been identified as a promising target for cancer immunotherapy (Jiang *et al*. 2020).

### 4.4 Gene-set co-enrichment

In addition, we used STRING to find the correlation between genes in the 50th, 70th, and 90th percentiles of DeepLIFT contribution scores and gene sets contained within STRING representing genes where protein products interact and form pathways. With 13 960 genes in our transcriptomics dataset, genes in the 50th, 70th, and 90th percentiles represent 6980, 4188, and 1396 genes, respectively, and genes in the different percentiles are independently assigned as input to STRING. To ensure proper analysis of enrichment sets and genes’ DeepLIFT scores, we filtered the enrichment sets with at least 30 genes.

After running our pretrained metalearning model from survival analysis on each target cancer type data, we sorted the genes by DeepLIFT scores and set the first gene from each enrichment set as the anchor gene. We then counted how many genes in the same enrichment set appear near the anchor gene in the sorted list. In this process, a standard for how near we look around the anchor gene becomes necessary, which we refer to as the window size. If a gene is within ± the window size from the anchor gene, we consider the two genes’ DeepLIFT scores to be similar. We set the window size to be the number of genes divided by 10, meaning that 698, 418, and 139 are the window sizes for the 50th, 70th, and 90th percentiles of genes, respectively.

On BLCA, with genes in the 50th, 70th, and 90th percentile of DeepLIFT scores, 18.8%, 19.7%, and 19.2% of genes in each enrichment sets had similar DeepLIFT scores, respectively, to the anchor gene in the sorted list. On the other hand, only 9.4%, 5.0%, and 2.4% genes were near the anchor gene in a randomly sorted list. Clearly, the more similar genes’ DeepLIFT scores, the more likely they were coenriched in functional sets. This indicates that using a metalearning model trained using Cox-loss on the transcriptomics genes, genes that are coenriched in the same enrichment sets were assigned similar DeepLIFT contribution scores. Since these enrichment sets are categorized by their functional attributions, this result indicates that the model captures functional and semantic relationships among genes without any prior knowledge. Results for each of the three cancer types are shown in Table 5.

**Table 5.** Genes in the same STRING enrichment sets are assigned similar DeepLIFT contribution scores

Genes sorted by DeepLIFT scores	Percentiles	No. of input genes	Window size	BLCA (%)	HNSC (%)	LUAD (%)
	50th	6980	698	18.8	19.2	19.2
Sorted	70th	4188	418	19.7	19.5	19.0
	90th	1396	139	18.8	21.3	20.0
	50th	6980	698	9.4	9.2	10.2
Random	70th	4188	418	5.0	6.0	5.6
	90th	1396	139	2.4	2.8	2.1

This is indicated by the percentages of genes from the same enrichment sets that are more likely to be clustered around the anchor gene in the sorted list in comparison to a randomly sorted list.

## 5 Conclusion

Although data integration and transfer of knowledge have been shown to be effective in other machine learning areas, omics data integration in comparison has only recently been explored considerably due to data sparsity, complexity, and extremely large feature space. Toward the goal of advancing omics data integration for survival analysis and pathway enrichment analysis, we used metalearning to show how multi-omics dataset integration improves cancer survival analysis. Whereas transcriptomics dataset is the most widely used, our results show that complementing it with clinical and proteomics datasets enhances performance of survival analysis. Furthermore, we combined metalearning with variable importance analysis methods of DeepLIFT, compared pathway enrichment using g: Profiler and GSEA, and compared enriched molecular pathways using the transcriptomics data alone and using integrated multi-omics data. Finally, we demonstrated how genes with similar, high DeepLIFT attribution scores are more likely to be coenriched in functional enrichment sets, which implies that training a meta-learning model can learn functional relationships of genes without prior knowledge.

*Financial Support:* None declared.

*Conflict of interest:* None declared.
